# Molecular docking analysis of Indole based oxadiazoles with the H-binding protein from *Treponema denticola*

**DOI:** 10.6026/97320630019079

**Published:** 2023-01-31

**Authors:** Poojitha Kumaran, Gayathri Rengasamy, Surya Sekaran, Kavitha Sankaran, Vishnu Priya Veeraraghavan, Rajalakshmanan Eswaramoorthy

**Affiliations:** 1 Department of Biochemistry, Saveetha Dental College and Hospitals, Saveetha Institute of Medical and Technical Science (SIMATS), Saveetha University, Chennai-600077, India; 2 Department of Biomaterials (Green lab), Saveetha Dental College and Hospital, Saveetha Institute of Medical and Technical Science (SIMATS), Saveetha University, Chennai-600077, India

**Keywords:** antimicrobial agent, diazole-thiol derivatives, Indole oxadiazoles, H-binding protein, *Treponema denticola*, In-silico

## Abstract

*Treponema denticola* is a gram-negative bacteria that is associated with periodontal diseases. Literature derived, six indole based oxadiazole derivatives are docked with the target Factor H binding protein (fHbp) protein. Results show better docking interaction compared to clinically proven drugs and all compounds obey Lipinski's rule of five. Hence, the compounds were inferred to be potential inhibitors for factor H binding protein of *Treponema denticola*.

## Background:

Periodontitis is one of the most common dental diseases caused mostly by infections and inflammation of the gums and bone that surround and support the teeth. In its early stages, known as gingivitis, the gums might become swollen, red, and bleed. Periodontitis is a more serious form of gum disease in which the gums peel away from the tooth, bone is lost, and teeth loosen or fall out. The two most serious dangers to oral health are periodontal disease and tooth decay [1]. Risk factors influence an individual's reaction to periodontal infection. Identification of these risk variables aids in the targeting of patients for preventive and therapy, with risk factor modification crucial to periodontal disease control. In the last several decades, changes in our knowledge of periodontal disease prevalence, as well as developments in scientific methods and statistical analysis, have enabled the discovery of numerous key systemic risk factors for periodontal disease [2, 3, 4]. *Treponema denticola* is a gram negative bacteria that is well associated with periodontal diseases. Factor H binding protein (fHbp) is a 27-kDa lipoprotein present on the surface that improves the survival of the bacterium in human blood by binding human factor H (hfH). T.denticola are also found in the natural oral flora of humans. They live mostly in the subgingival region because they are anaerobic. They may, however, take hold in opportunistic infections such periodontal diseases, which are damaging, inflammatory processes of the tooth attachment tissues caused by gram-negative anaerobic proteolytic bacteria [3]. Different spirochetal morphotypes can be seen in periodontal pockets, however many of these morphotypes have yet to be identified as uncultivable 6]. One of the most extensively researched oral microorganisms, T.denticola exhibits the required characteristics for periodontal tissue adhesion, invasion, and damage [2]. Therefore, researchers in the field of antimicrobial chemotherapy are trying to search and explore novel drugs to decrease the risk. Chemo-informatics developments have resulted in the creation of virtual chemical libraries that may be screened. Furthermore, computer approaches for predicting the drug-likeness of molecules are being developed [8]. Therefore, it is of interest to document the molecular docking analysis of Indole based oxadiazoles with the H-binding protein from *Treponema denticola*.

## Materials and methods

## Preparation of ligands:

**Figure 1:**
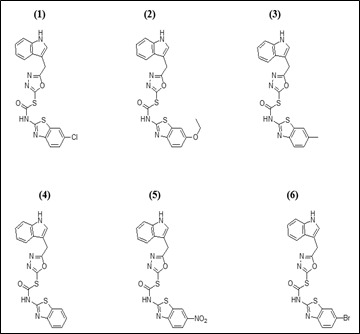
2D Structures of the diazole-thiole compounds (1-6)

The 2D structures of the selected diazole-thiole compounds were drawn using Chem Draw 16.0 (Figure 1). During the optimization method, the software Chem3D was employed and all parameters were selected in order to achieve a stable structure with the least amount of energy. The structural optimization approach was used to estimate the global lowest energy of the title chemical. Each molecule's 3D coordinates (PDB) were determined using optimized structure.

## Preparation of macromolecules:

**Figure 2:**
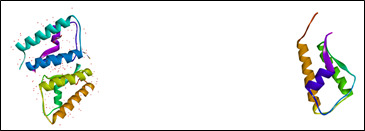
3D structure of factor H-binding protein of *Treponema denticola*

The 3D crystal structure of the factor H binding protein of *Treponema denticola* (PDB ID: 3qz0) was downloaded from the protein data bank (Figure 2). As per standard protocol, protein preparation was done. Water molecules, co-crystallized ligands, and other cofactors were chosen for elimination. The protein structure was constructed by adding polar hydrogens and Kollman charges with Auto Prep [7].

## Auto dock Vina analysis:

**Figure 3:**
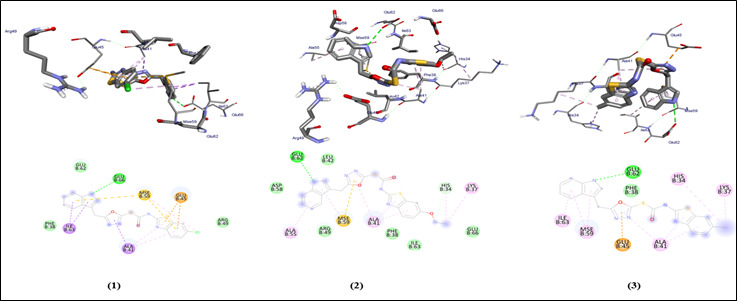
Molecular docking analysis of compounds (1-3) against H binding protein of *Treponema denticola*

**Figure 4:**
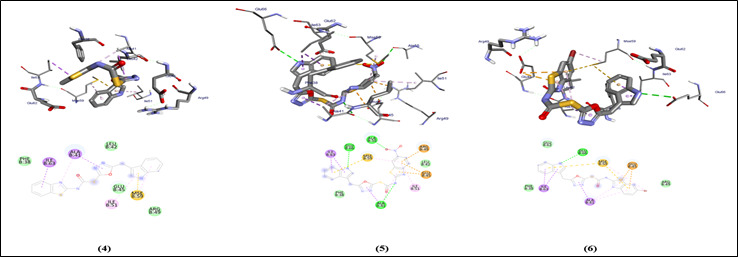
Molecular docking analysis of compounds (4-6) against H binding protein of *Treponema denticola*

The graphical user interface Auto Dock vina was used for Ligand-Protein docking interactions (Figure 3-Figure 4). Auto Dock Tools (ADT), a free visual user interface (GUI) for the AutoDock Vina software, was used for the molecular docking research. The grid box was built with dimensions 25.0, 13.3823, 16.630 A pointing in the x, y, and z axes. The central grid box for 3QZ0 was 10.3156, 23.9999, 44.4203 A. For each ligand, nine alternative conformations were created and ranked based on their binding energies utilizing Auto Dock Vina algorithms.

## In-silico drug-likeness and toxicity predictions:

SwissADME and PROTOX-II online servers were used. This prediction points users in the direction of drug efficiency and gives information on whether or not the examined ligand has features consistent with becoming an orally active medication. This prediction is based on Lipinski et al's previously established idea known as Lipinski's rule of five [9].

## Statistical analysis:

One way ANOVA was used for statistical analysis. The clinically proven drugs are used as control and the results are compared. The significance of the results was found to be p < 0.05.

## Results:

## Molecular interaction against factor H binding protein (3qz0):

All compounds run against the protein have binding energy in the range of -6.9 to -7.3 Kcal/mol (Table 1). The compounds show a H binding interaction similar to that of sulfamethoxazole (-5.6). Clinically proven drugs shown in lead binds to the binding site of protein. Azithromycin binds to the ALA-41 binding site. Sulfanilamide binds to the LYS-37, MSE-59 and GLU-62 binding sites. Sulfamethoxazole binds to the ALA-55 binding site of the protein. All compounds show similar binding affinity as the lead molecules within the binding site.

## ADME and Lipinski rule of five:

Pharmacokinetic properties (ADME), drug-likeness, toxicity profiles are examined using SwissADME, and ProTox-II online servers. The SwissADME, a web tool from Swiss Institute of Bioinformatics (SIB) is used to convert the two-dimensional structures into their simplified molecular input line entry system (SMILES). The physicochemical properties (molar refractivity, topological polar surface area, number of hydrogen bond donors/ acceptors); pharmacokinetics properties (GI absorption, BBB permeation, P-gp substrate, cytochrome-P enzyme inhibition, skin permeation (log Kp)) which are critical parameters for prediction of the absorption and distribution of drugs within the body, and drug likeness (Lipinski's rule of five) were predicted using SwissADME (Table 2 and Table 3).

## Toxicity profile (ADMET):

The toxicological endpoints (hepatotoxicity, carcinogenicity, immunotoxicity, mutagenicity) and the level of toxicity (LD50, mg/Kg) are determined using the ProTox-II server. The compounds show class 4 toxicity and have inactive immunotoxicity, cytotoxicity and mutagenicity (Table 4). The lethal dose parameters of the chosen molecules are lesser than the control group and thus can be used as drugs. The chosen compounds show a toxicity profile similar to that of the control drug, sulfamethoxazole.

## Discussion:

Azithromycin in a combined form with hydroxychloroquine (HCQ-AZ) was administered in several clinics as a potential drug for the treatment of COVID19. It was inferred that Azithromycin showed a good affinity with the target proteins. It is also known to act as a good inhibitor to use against the acute respiratory coronavirus 2 [12]. Sulfanilamide is an effective antibacterial drug that functions by inhibiting the para-amino benzoic acid (PABA) [13]. Sulfamethoxazole is an antibiotic used for the treatment of infections like prostatitis and other urinary tract infections. It is also effective against both gram positive and gram-negative bacteria [14]. Results from the molecular docking studies infer that the compounds (1-6) show higher affinity i.e., the binding score with the protein binding sites is lesser (Table 1). In (Table 2 and Table 3) the SWISS-ADME tool was used to evaluate the drug-likeness of a compound by Lipinski’s rule of five. This ensures that the drug is consistent with the properties of an orally active drug [5]. All the chosen compounds, (1-6) follow Lipinski's rule of 5 and thus can be used as active oral drugs. The control compound, Azithromycin, shows 2 violations of the rule. The LD50 value of the compound and the toxicity profile are shown in (4). The toxicity profile of the chosen compounds is similar to the control group compound, sulfamethoxazole. The LD50 values of the compounds were also better than the control group, Azithromycin. The chosen compounds show a logKp value within the range of -5.89 to -5.26 cm/surface. It is inferred that the more the negative value, the higher will be the permeation of skin. All the compounds show lesser negative values of log Kp than the lead compounds. The GI absorption of the compounds is low and thus will need a carrier molecule to perform its function. This is similar to the control compound Azithromycin. The chosen compounds do not show any blood brain barrier permeability. The control group taken does not inhibit the CytP and the compound 5 is similar in function to the control group, Azithromycin. Therefore, the compound 5 can be used as a potential drug. All compounds obey Lipinski's rule of five and are similar to the control groups taken. Additionally, the control group molecule Azithromycin shows violation in the molecular weight (748.9) and TPSA (180.08). In spite of these exceptions, this can still be used as a drug as these violations don't directly affect its function. Compared to this, the chosen compounds don’t have any violations and therefore can be used as lead molecules.

## Conclusion:

The selected diazole-thiole derivatives show better docking interaction compared to clinically proven drugs and all compounds obey Lipinski's rule of five. The compound 5 shows similar characteristics to that of the control group drugs and the binding affinity value of it is -7.1 which has more negative score compared with that of the clinically proven ones. Thus, the compound 5 can be a potential drug against factor-H binding protein of *Treponema denticola*.

## Figures and Tables

**Table 1:** Molecular docking interaction of the diazole-thiole compounds (1-6) against factor H binding protein of *Treponema denticola* (PDB ID: 3qz0).

Ligands	Docking scores/Affinity (kcal/mol)	H-bond	Amino Acid Residual interactions	
			Hydrophobic/Pi-Cation	Van dar Waals
1	-7.1	GLU-66	ILE-63, ALA-41, MSE-59, GLU-45	ARG-49, GLU-62, PHE-38
2	-7	GLU-62	MSE-59, ALA-41, HIS-34, LYS- 37, ALA-55	ASP-58, ARG-49, LEU-42, PHE-38, ILE-63, GLU-66
3	-6.7	GLU-62	ILE-63, MSE-59, GLU-45, ALA-41, LYS-37, HIS-34	PHE-38
4	-6.9		ILE-63, ALA-41, ILE-51, MSE-59	PHE-38, ARG-49, GLU-45, LEU-42
5	-7.1	GLU-66, ALA-55, ALA-41	ILE-63, MSE-59, ARG-49, GLU-45, ILE-51	LEU-42, PHE-38
6	-7.3	GLU-66	MSE-59, GLU-45, ALA-41, ILE-63	GLU-62, ARG-49, PHE-38
Azithromycin	3.8	ALA-41	MSE-59, GLU-66	ALA-55, LEU-42, ARG-49, ILE-51, GLU-62, GLU-45, ASN-44, LYS-37, HIS-34, ILE-63, PHE-38
Sulfanilamide	-4.4	LYS-37, MSE-59, GLU-62	ILE-63	GLU-66, ALA-41, HIS-34
Sulfamethoxazole	-5.6	ALA-55	MSE-59, LYS-37, ILE-63, ALA-41	LEU-42, GLU-45, PHE-38

**Table 2:** SwissADME values of selected diazole-thiole compounds (1-6)

Compound	log Kp (cm/s)	GI absorption	BBB permeant	Pgp substrate	CYP1A2 inhibitor	CYP2C19 inhibitor	CYP2C9 inhibitor	CYP2D6 inhibitor	CYP3A4 inhibitor
1	-5.26	Low	No	No	Yes	Yes	Yes	Yes	Yes
2	-5.53	Low	No	No	No	Yes	Yes	Yes	Yes
3	-5.32	Low	No	No	No	Yes	Yes	Yes	Yes
4	-5.49	Low	No	No	Yes	Yes	Yes	Yes	Yes
5	-5.89	Low	No	No	No	Yes	No	No	Yes
6	-5.48	Low	No	No	Yes	Yes	Yes	Yes	Yes
Azithromycin	-8.01	Low	No	Yes	No	No	No	No	No
Sulfanilamide	-7.79	High	No	No	No	No	No	No	No
Sulfamethoxazole	-7.21	High	No	No	No	No	No	No	No

**Table 3:** Lipinski and Veber rules of selected diazole-thiole compounds (1-6)

Compound	MW	iLogP	HBD (nOHNH)	HBA (nON)	nrotb	MR	TPSA	Lipinski #violations	Bio availability score
Lipinski*	≤500	≤5	≤5	≤10	≤10	-	-		
Veber**	-	-	-	-	-	-	≤ 140		
1	441.91	3.09	2	5	6	114.93	150.24	0	0.55
2	451.52	3.03	2	6	8	121.22	159.47	0	0.55
3	421.5	3.1	2	5	6	114.89	150.24	0	0.55
4	407.47	2.71	2	5	6	109.92	150.24	0	0.55
5	452.47	2	2	7	7	118.74	196.06	0	0.55
6	486.36	3.16	2	5	6	117.62	150.24	0	0.55
Azithromycin	748.98	4.76	5	14	7	200.78	180.08	2	0.17
Sulfanilamide	172.2	0.61	2	3	1	41.84	94.56	0	0.55
Sulfamethoxazole	253.28	1.03	2	4	3	62.99	106.6	0	0.55

**Table 4:** Toxicity profile of selected diazole-thiole compounds (1-6)

Compound	^a^LD_50_ (mg/kg)	Class	Toxicity				
			Hepatotoxicity	Carcinogenicity	Immunotoxicity	Mutagenicity	Cytotoxicity
1	1000	4	Active	Active	Inactive	Inactive	Inactive
2	1000	4	Active	Active	Inactive	Inactive	Inactive
3	1000	4	Active	Active	Inactive	Inactive	Inactive
4	1000	4	Active	Active	Inactive	Inactive	Inactive
5	1000	4	Active	Active	Inactive	Inactive	Inactive
6	1000	4	Active	Active	Inactive	Inactive	Inactive
Azithromycin	2000	4	Inactive	Inactive	Active	Inactive	Inactive
Sulfanilamide	3000	5	Inactive	Active	Inactive	Inactive	Inactive
Sulfamethoxazole	2300	5	Active	Active	Inactive	Inactive	Inactive
^a^LD_50_: lethal dose parameter
